# Influences of Indoor Air Temperatures on Empathy and Positive Affect

**DOI:** 10.3390/ijerph21030323

**Published:** 2024-03-10

**Authors:** Rania Christoforou, Hannah Pallubinsky, Tobias Maria Burgholz, Mahmoud El-Mokadem, Janine Bardey, Kai Rewitz, Dirk Müller, Marcel Schweiker

**Affiliations:** 1 Healthy Living Spaces Lab, Institute for Occupational, Social and Environmental Medicine, Medical Faculty, RWTH Aachen University, 52074 Aachen, Germany; 2 Department of Nutrition and Movement Sciences, NUTRIM School of Nutrition and Translational Research in Metabolism, Faculty of Health, Medicine and Life Sciences, Maastricht University, 6211 KL Maastricht, The Netherlands; 3 Institute for Energy Efficient Buildings and Indoor Climate, E.ON Energy Research Center, RWTH Aachen University, 52074 Aachen, Germany; 4 Heinz Trox Wissenschafts gGmbH, 52074 Aachen, Germany

**Keywords:** cool, warm, social connection, empathy, thermal sensation, skin temperature

## Abstract

The consequences of climate change are already visible, and yet, its effect on psychosocial factors, including the expression of empathy, affect, and social disconnection, is widely unknown. Outdoor conditions are expected to influence indoor conditions. Therefore, the aim of this study was to investigate the effect of indoor air temperature during work hours on empathy, positive and negative affect, and social disconnection. Participants (*N* = 31) were exposed, in a cross-over design, to two thermal conditions in a simulated office environment. Questions on empathy and social disconnection were administered before and after the exposure to each condition, while affect was measured throughout the day. Subjective thermal sensation and objective measures of mean skin temperature were considered. The results indicated a significant difference in empathy (*F*(1, 24) = 5.37, *p* = 0.03, with an *η*^2^ = 0.126) between conditions. Participants reported increases in empathy after exposure to the warm condition compared to the cool condition, in which reductions in empathy were reported. Although the same pattern was observed for positive affect, the difference was smaller and the results were not significant. Thermal sensation had a significant effect on changes in empathy too (*F*(1, 54) = 7.015, *p* = 0.01, with an *R*^2^ = 0.115), while mean skin temperature had no effect on empathy (*F*(1, 6) = 0.53, *p* = 0.89, with an *R*^2^ = 0.81). No effects were observed for positive and negative affect and social disconnection. Longitudinal studies are needed to support these findings.

## 1. Introduction

There is an overwhelmingly body of evidence showing that our planet’s climate has been changing since the pre-industrial period [1,2]. Historical data show that Earth has been warming up and it is expected that global air temperatures will rise by up to 4 °C above pre-industrial temperatures by 2100 [3], changing both indoor and outdoor air temperatures. Extreme weather events, such as heatwaves, droughts, and wildfires, have been increasing in frequency, raising major concerns about the future of ecosystems and the impact this will have on human beings [2].

A large part of the world’s population is forced to live and work in thermal conditions that are outside their comfort zone. Due to global warming and the lack of infrastructure to cope with increasing temperatures in some European countries (e.g., in Germany, where the majority of buildings are not equipped with air-cooling systems), people experience warmer thermal conditions indoors than in earlier years. These changes in indoor air temperatures highlight the need to investigate the impact that these thermal conditions have on people, especially due to the fact that people spend the majority of their time indoors (approximately 90% of their day [4]).

### 1.1. Current Evidence on the Impact of Indoor Air Temperatures

Studies investigating the effects of the indoor environment on occupants suggest that indoor environmental conditions contribute significantly to human health, risk of infection, work performance, and cognition [5]. Factors associated with indoor environmental conditions include the air quality of a space, in addition to visual, acoustical, and thermal conditions and their interactions [6]. However, for the purpose of this paper, emphasis will be put on the thermal conditions, which are anticipated to be one of the most affected indoor factors by climate change especially due to more extreme outdoor temperature events. This hypothesis can be assumed to be particularly true for regions whose building stock has only a low proportion of air conditioning, but which are nevertheless already increasingly affected by extreme heat events [7]. For example, in Germany, only 2% of residential buildings and 33% of non-residential buildings are air-conditioned [8]. Consequently, the changes in outdoor temperature will lead to an effect on indoor temperature for those kind of buildings, although it is forecast that the proportion of air-conditioned buildings in Germany will increase [9].

High and low indoor temperatures have been associated with multiple negative health effects in numerous studies. In particular, high indoor temperatures have been associated with respiratory difficulties, diabetes management, core schizophrenia, and dementia symptoms [10]. Reported indoor temperatures associated with the worsening of these health issues range between 26 °C and 32 °C [10]. Additionally, cooler conditions have been associated with health difficulties, such as cardiovascular and mental health problems [11,12]. Furthermore, several studies indicate positive health effects after repeated exposure towards both (mild) cold and heat, reporting an enhanced thermoregulatory capacity and positive health effects from repeated thermal stress. For example, Hanssen et al. [13] showed that repeated exposure to mild cold (~14 °C) significantly enhanced metabolic health in patients with type 2 diabetes. Moreover, Pallubinsky et al. [14] suggested that repeated heat exposure (~34 °C), resulting in acclimation, and thus increased resilience towards heat, could also positively influence metabolic health. These findings raise the question of whether higher and lower temperatures than are currently considered appropriate for maintaining a positive health status could also have a positive effect on building occupants.

### 1.2. Indoor Air Temperatures and Psychosocial Factors

According to a large longitudinal research project, which used multiple methods to evaluate the heath impact of increasing energy performance in households, increasing energy efficiency and modifying the thermal conditions by approximately 1 °C during the winter period did not have a significant effect on cardiorespiratory emergency hospital admissions nor mental health [15]. However, increasing the indoor air temperatures by 1 °C had a significant effect on reducing the reluctance of occupants to invite others to their homes, and hence, promoting more social connection. The importance of focusing on social connection has gained visibility, especially after the COVID-19 pandemic, a period that demonstrated the detrimental effects that social isolation can have on physical and mental health [16]. Improving conditions to encourage social connection could potentially improve physical and mental health in the long-term. In the Poortinga, Rodgers, Lyons, Anderson, Tweed, Grey, Jiang, Johnson, Watkins, and Winfield’s [15] study, the change in social behaviour was attributed to the fact that people felt comfortable using more spaces in their homes because of the warmer thermal conditions. Nevertheless, other studies suggest that temperature can influence psychosocial behaviours via other pathways too, which were not taken into consideration in the Poortinga, Rodgers, Lyons, Anderson, Tweed, Grey, Jiang, Johnson, Watkins, and Winfield [15] study, such as the shared neural pathway between physical and social warmth [17].

In general, previous studies have supported a bidirectional relationship between temperature and psychosocial variables, although less emphasis was given to the impact of indoor air temperature on psychosocial factors. Studies have argued that there is a shared neural mechanism underlying physical and social warmth and hence modification of either temperature or social connection will probably lead to a change in the other [18]. This shared neural pathway was supported in other studies too, which found that people who were feeling physically cold also felt significantly more lonely than individuals experiencing physical warmth [19]. Consequently, this shared mechanism could be another explanation for how indoor air temperature could influence psychosocial aspects via changes in peripheral body temperatures.

Taking into consideration this shared neural pathway and the outcomes from previous studies, one might expect that increasing indoor temperatures might be beneficial for occupants, at least with regard to social connection. However, there are also studies supporting a negative association between indoor temperatures and prosocial behaviours. For example, Kolb, Gockel, and Werth [20] reported that, when participants were exposed to lower indoor temperatures, they demonstrated more prosocial behaviours (i.e., gave more discounts to their customers) compared to when they were exposed to higher indoor air temperatures. This negative association could be explained by the social thermoregulation theory, which suggests that people seek more social connection when they are feeling cold in order to regulate their body temperature [21]. Another example is the notion that higher temperatures can lead to increased levels of aggression and negative affect as reported by Anderson, Deuser, and DeNeve [22]. Although the association between temperature and aggressive behaviours has been repeatedly established, the majority of previous studies to our knowledge used games or words [23] that could trigger this aggression. Therefore, moderating effects might have masked the actual effects of indoor temperatures on prosocial behaviours and positive affect. One of the studies that investigated the effect of outdoor air temperature on aggressive behaviour in the form of assaults supported a positive relationship between temperature and number of assaults when taking into consideration the time of the day, since assaults are more likely to happen at night [24]. However, this study did not control for the presence of any mental health difficulties reported in the sample taken into consideration. Recent studies investigating the effect of outdoor ambient temperature on mood suggest that individuals with specific mental health difficulties are more prone to negative affect, and potentially to acts of aggression, than individuals with no mental health difficulties when there is an increase in ambient temperature [25]. According to Bundo, Preisig, Merikangas, Glaus, Vaucher, Waeber, Marques-Vidal, Strippoli, Müller, and Franco [25], this could be due to the fact that those people might have less opportunities for social activities, which become more prominent under rising temperatures, a weather state that encourages social activities and social connection. Therefore, their acts of aggression might stem from the negative affect associated with their social isolation. This contradiction in outcomes and the numerous moderating factors indicate that further research is needed in controlled environments with healthy participants before concluding on the effect that higher and lower indoor air temperatures can have on psychosocial factors in the future.

### 1.3. Specific Psychosocial Factors of Interest

In this study, we are particularly interested in changes reported in social connection, affect, and empathy, since these three factors play a major role in developing and maintaining mental health difficulties [26,27,28,29]. People are undeniably considered to be “social beings”. We are born with the help of others, we need others to survive, and we feel lonely when social connection is not present [30]. Short-term feelings of loneliness (i.e., social disconnection) are experienced by everyone, but long-term feelings of loneliness can lead to serious mental health difficulties, such as depression [30]. Depression increases the risk of suicidality [31]. Therefore, being able to predict potential increases in depression and suicidality in order to apply preventive measures is of primary importance. Additionally, the expression of emotions is reported by Bruno, Melnyk, and Völckner [32] to be a potential mechanism for maintaining homeostasis (i.e., balance) in our physiological reactions. Consequently, understanding the changes in our emotions and affect during temperature exposure could enhance our understanding of the influence of temperature conditions on psychopathology. Furthermore, the way positive and negative feelings influence how we interact with others and our environment, and potentially our mental health, highlights the urgency of focusing on changes in affect triggered by the changing temperatures [33]. Finally, empathy, which is the ability to cognitively and affectively perceive the emotions of others, has been associated with people’s ability to connect with other people, receive support, and feel understood [34]. Empathic responses have been found to aid the recovery process from both physical and mental health difficulties [35]. Therefore, investigating changes in empathic responses, triggered by extreme temperatures, could inform future prediction models for the development and maintenance of mental health problems within the population. To our knowledge, there are currently no studies investigating the effect of indoor air temperatures on these psychosocial variables.

### 1.4. The Effect of Indoor Air Temperature at Work on Psychosocial Factors

Another gap in the literature is how indoor temperature, specifically in non-residential work buildings, could influence psychosocial factors, considering that many people, who work full-time, spend the majority of their awake time at work. The effects for situations when working remotely at other environments are not considered here. To our knowledge, so far, studies have only addressed the effect of indoor temperature on psychosocial factors in residential settings [15]. Studies focusing on occupational settings quite often collect data on psychosocial factors, but the association between indoor temperature and psychosocial factors is missing [36]. Consequently, focusing on non-residential occupational environments can aid the development of recommendations for work environments, which could potentially be implemented as a preventive measure against the challenges faced due to climate change or to help people adapt to the changing indoor environmental conditions.

### 1.5. Current Study

The current study focuses on the psychological effects of a larger project investigating the effects of indoor temperature at work on physiological and psychological aspects. More specifically, the primary aim of the present study was to investigate the effects of thermal indoor conditions on social disconnection, empathy, and the positive and negative affect of regular office workers in a simulated office environment. A secondary aim was to investigate the effect of thermal sensation and mean skin temperature on the same psychosocial factors, to observe which of the two might be a better predictor of the effect that indoor thermal conditions can have on psychosocial aspects. Based on the notion that physical and social warmth share a common neural pathway and that social activation was observed with increased temperatures in residential contexts, it was hypothesized that:

1. A significant difference will be observed between the effect of the warm and the cool condition on levels of social disconnection, empathy, positive and negative affect, i.e., participants will report more empathy and positive affect in the warm condition than in the cool condition, and less social disconnection and negative affect in the warm vs. the cool condition.
2. A significant effect of thermal sensation on social disconnection, empathy, positive and negative affect will be observed, i.e., participants perceiving the environment as warm or hot will report more empathy, more positive affect, less social disconnection, and less negative affect than when they experience the environment as cool or cold.
3. A significant effect of mean skin temperature on social disconnection, empathy, positive and negative affect will be observed, i.e., higher levels of empathy and positive affect, and lower levels of social disconnection and negative affect will be reported when the mean skin temperature of the participants is higher.

## 2. Materials and Methods

### 2.1. Participants

An a priori sample size calculation (G*Power: Wilcoxon signed-rank test (matched pairs), one-tailed, *α* = 0.05, *β* = 0.80, *η*^2^ = 0.5—medium effect size) led to a recommended minimum sample size of 28 participants. However, an additional 15% of participants was considered to account for potential dropouts. A sample of 32 participants was recruited. One of the participants was not able to complete the study for personal reasons. Therefore, a sample of 31 self-reported healthy participants (females: 16, males: 15, average age: 40.7 ± 16.5 years, 90% German) was included in the analyses. The sample consisted mainly of German participants (*N* = 28). There were also participants from Belgium (*N* = 1), Portugal (*N* = 1), and Greece (*N* = 1). Most of the participants were students (*N* = 14) and the rest were employed in a variety of sectors (e.g., three in medicine, one participant in each of the following: architecture, engineering, research, law, psychology, and eight from other fields).

### 2.2. Procedure and Ethical Considerations

Ethical approval for this study was granted by the ethics committee of the medical department at RWTH Aachen University. Participants were recruited via convenience sampling from participant lists, snowball sampling, and via advertisements on social media platforms, local newspapers, and local amenity shops during summer 2021. Before participating in this study, each participant was asked to complete a series of questions, which formed a pre-screening assessment to ensure that participants were fulfilling the inclusion criteria. Participants were excluded from the study if they had: (i) a current diagnosis of COVID-19 at the time of data collection, (ii) a medical condition that could influence outcomes in terms of thermoregulation and/or cognitive performance either directly via the condition or indirectly via the prescribed medication, (iii) fluctuations in body weight as an indirect indication of potential health issues, (iv) high alcohol consumption (i.e., for males: >2 servings per day, for females: >1 serving per day), and (v) regular smoking habits for the last 12 months before the study. Additional exclusion criteria for women included pregnancy and unstable post-menopausal stage in the older age group, since these could interfere with the study measurements. Based on the aforementioned criteria, 39 individuals were excluded from the study in the pre-screening phase.

**Figure 1:**
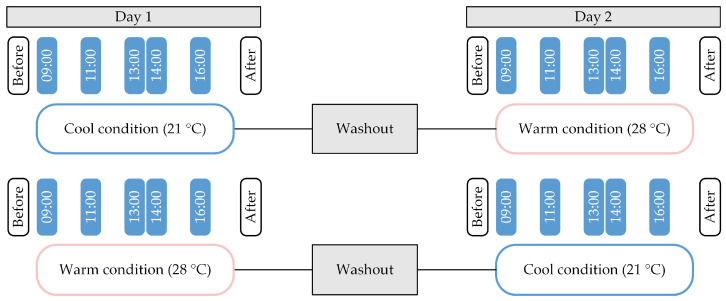
Representation of the cross-over study design and of data collection points for subjective measures. Questionnaires assessing empathy and social disconnection were administered before and after the exposure. Questionnaires on the perception of environmental conditions (thermal sensation) and related affect were administered at 09:00 for baseline assessment and then twice in the morning (at 11:00 and 13:00) and twice in the afternoon (at 14:00 and 16:00) with a two-hour gap in between. All objective measurements (monitoring of environmental conditions and skin temperature) were assessed every minute throughout the day.

**Figure 2:**
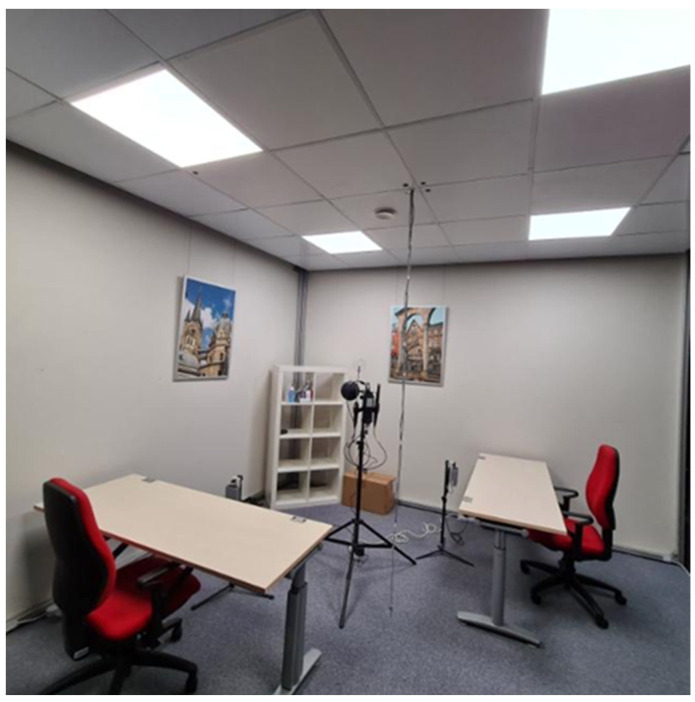
A photo of the laboratory environment, where the participants were asked to stay between 09:00 and 17:00. In the middle of the room, a Testo sensor is monitoring the indoor temperature conditions and humidity. In front of each desk, there are the non-commercial, tailor-made sensors measuring air temperature and humidity for each participant.

All eligible participants were invited to attend two testing days, which followed the same hybrid format (laboratory and field study) under two different conditions (a cool condition: ~21 °C, and a warm condition: ~28 °C). The thermal conditions were chosen based on a priori calculations based on the Fanger’s predicted mean vote (PMV) model [37] using the R package comf [38]. Participants were asked to wear clothing related to around 0.5–0.6 clo according to table values of ISO 7730 [39]. Metabolic rate for sedentary office work was set to 1.2 met according to table values of ISO 7730. Assuming minimal air velocity (<0.05 m/s) and a relative humidity of 50%, we set the air and radiant temperature of the two conditions to 21 °C and 28 °C, which resulted in a PMV of −1 (slightly cool) and +1 (slightly warm), respectively. These conditions were chosen as they are distinct enough to expect an effect, while still within a range acceptable for a full working day. Participants were exposed to the two conditions in a cross-over design (see Figure 1). Before each testing day, participants were asked to report in a diary the activities performed the day before the exposure and their food intake, and they were asked to repeat the same activity and food schedule before the second experimental day to keep the conditions as similar as possible. Upon arrival to the testing days, participants were tested for COVID-19, if they had no proof of vaccination or negative test with them, and if they were negative, they were equipped with sensors to record their skin temperature (iButtons^®^, Maxim Integrated, Munich, Germany, Accuracy: ±0.5 K, Resolution: ±0.0625 K) for 24 h (08:00 in the morning of testing day until 08:00 in the morning of the following day). For each testing day, participants were asked to work on an activity of their choice among typical office tasks in a simulated office environment (lab—see Figure 2) for eight hours (09:00–17:00 to mimic typical work), while being equipped with the above-mentioned sensors and answering a series of questionnaires (see measures section), with a follow-up field assessment at their home (field data are beyond the scope of this paper). Although the work-related activities performed by the participants were not restricted by the experimenters in order to ensure a realistic work-environment content, the experimenters reported the activities performed. To ensure the washout of any effects from the preceding condition, a gap of at least one day was maintained between the two experimental days for each participant. Before being exposed to any conditions, participants were informed about the study and an informed consent was obtained from them. The exact aim of the study was not revealed to them until the end of their participation.

Indoor environmental factors within the simulated environment, such as visual, acoustic, absolute air humidity, and air quality aspects, were controlled and maintained constant throughout the study, as much as possible. Only indoor temperature varied according to the condition allocated to each testing day. Measures assessing indoor environmental factors, the perception of the participants of the indoor environment, and related affect, were administered throughout the day, while measures on social disconnection and empathy were only administered before and after exposure to the different thermal environments, for both conditions (see Figure 1). Participants were asked to wear similar, preferably identical, clothes for both conditions (long-sleeved light top or short-sleeved light top, long trousers, underwear, socks, and low shoes) to minimize variance due to the effect of clothing on thermal perception and comfort between the two conditions.

### 2.3. Measures

#### 2.3.1. Demographic and Participant Characteristics

Demographic characteristics were collected, such as participants’ sex (male or female), year of birth, nationality, and employment status. Additional participant characteristics were assessed, such as participants’ personality characteristics, emotion regulation strategies, levels of alexithymia, and self-efficacy, to provide a psychological profile of the sample.

Firstly, participants’ personality characteristics were assessed using a short version of the 44-item Big-Five Inventory [40] in the German language (BFI-10 [41]). The BFI-10 consists of 10 items, which are rated on a 5-point Likert scale from 1 (Strongly disagree) to 5 (Strongly agree). Higher scores indicate higher levels of the subscale theme (i.e., of extraversion, agreeableness, conscientiousness, neuroticism, openness). The German BFI-10 has demonstrated moderate test-retest reliability (*r* = 0.49–0.84 [41]).

Secondly, two emotion regulation strategies used by the participants were evaluated using the German version of the Emotion Regulation Questionnaire (ERQ—German [42]): (i) cognitive reappraisal, a subscale within the ERQ, in which higher scores mean that the participants prefer to reassess a situation and give a different meaning to it to cope with the emotional stress of a situation (i.e., actively cope with a situation), and (ii) expressive suppression, another subscale of the ERQ, in which higher scores mean that the participant prefers to inhibit or restrict the inner emotions to cope with a situation (i.e., avoid a situation/feeling). The ERQ consists of 10 items, rated on a 7-point Likert scale from 1 (Not true at all) to 7 (Completely true). From the total 10 items, 6 items belong to the cognitive reappraisal scale and 4 to the expressive suppression scale. The German ERQ has demonstrated good validity and reliability (Cronbach’s alpha: 0.74–0.76 [42]).

Thirdly, participants’ levels of alexithymia (i.e., difficulty to identify and describe their emotions) were assessed using the German version of the Toronto Alexithymia Scale (TAS-20 [43]). TAS-20 consists of 20 items, rated on a 5-point Likert scale from 1 (Strongly disagree) to 5 (Strongly agree). Total scores can range between 20 and 100. Higher scores indicate higher levels of alexithymia. The German version of the TAS-20 demonstrated good internal consistency and adequate reliability and validity [43].

Lastly, the self-efficacy of the participants was assessed using a German scale investigating general self-efficacy belief, the “Allgemeine Selbstwirksamkeitserwartung” (SWE) questionnaire [44]. SWE consists of 10 items, rated on a 4-point Likert scale from 1 (Not true) to 4 (Exactly true). The total score of SWE is calculated by adding all the item scores together. Higher scores indicate greater belief on self-efficacy. The SWE has shown good reliability (Cronbach’s alpha scores: 0.71–0.89 [44]).

#### 2.3.2. Indoor Environmental Factors

Indoor environmental factors were assessed using a non-commercial, tailor-made sensor measuring air temperature and humidity, which also made it possible to measure data in the field at participants’ homes. Minute data of indoor air temperature and humidity were digitally collected and analysed after calibration. The non-commercial, tailor-made sensors were calibrated by comparing the values with a commercially available sensor (Testo 480 (0563-4800), Probe 0632 1543, Titisee-Neustadt, GER), which was also present within the simulated office environment during the laboratory part of the study.

#### 2.3.3. Thermal Sensation

Thermal sensation data were collected using one of the most prominent items for thermal sensation, listed in the ASHRAE 55 standard scales [45]. In this item, participants had to report how cold or hot they sensed the environment on a 7-point scale. For the purposes of this study, only the mean scores of each participant during the laboratory part of the study were taken into consideration for each testing day separately. This included the six votes collected between 09:00 and 18:00 of each testing day (before the exposure, twice in the morning, twice in the afternoon and after the exposure as indicated in Figure 1).

#### 2.3.4. Skin Temperature

Skin temperature was recorded continuously for each participant using iButtons^®^, wireless skin temperature sensors. iButtons were placed at the four ISO-defined sites [46]. Minute data were collected for the whole duration of the testing day. However, for the purposes of this study, the mean skin temperature during the laboratory part of each testing day (i.e., 09:00–17:00) was calculated and taken into consideration. A calibration of the sensors was conducted before analysing the results.

#### 2.3.5. Social Disconnection

Feelings of social disconnection were assessed using a 5-item scale used in a previous study by Eisenberger et al. [47]. The items were rated on a 5-point Likert scale from 1 (Not at all) to 5 (Very strongly) and included questions on how close the participants felt to other people and how close they wanted to be with others. Higher scores indicated stronger feelings of social disconnection, i.e., reduced social connection.

#### 2.3.6. Empathy

Empathy levels were assessed using three self-report scenario questions. The scenarios assessed the empathic responses of the participants in three different situations: (i) returning home after work and finding their partner/family member having a bad day, (ii) returning home from work and finding out that their partner/family member had to stay longer at work because of a deadline, missing important plans for the afternoon, and (iii) returning home from work and finding their partner/family member lying on the sofa/bed looking very ill. Each answer was independently scored with values from 0 to 2 depending on the empathic response. A total score was calculated by summing the scores for all three scenarios together. Higher scores indicate higher levels of empathic responses. The scenarios were developed for the purposes of this study based on scenarios previously used and validated with different population samples [48].

#### 2.3.7. Positive and Negative Affect

Positive and negative affect was assessed using one of the most widely used measures of affect, the Positive and Negative Affect Schedule (PANAS) scale in the German language [49]. The PANAS scale consists of 20 items describing different feelings and perceptual aspects. Participants were asked to rate each feeling on a 5-point scale from 1 (Not at all) to 5 (Extremely). The PANAS scale is separated into two subscales: (i) the positive affect subscale (10 items), and (ii) the negative affect subscale (10 items). The score for each subscale is the mean score of its items. Higher scores on the positive affect subscale represent greater levels of positive affect and higher scores on the negative affect subscale represent greater levels of negative affect. The PANAS scale in the German language has demonstrated very good validity and reliability (Cronbach’s alpha = 0.86 [49]).

### 2.4. Statistical Analysis

For the statistical analyses the R Studio (version 2023.03.01 [50]) was used. The total scores of the social disconnection, empathy, positive and negative affect scales for each data collection point were calculated for each participant. Afterwards, differences in the total scores reported between “after the thermal exposure” and “before the thermal exposure” were calculated and were used in addition to the mean scores of each testing day for thermal sensation and skin temperature for the analyses. To test Hypothesis 1, the data were separated by the condition that participants were exposed to (21 °C and 28 °C). Normality tests were conducted on the variables of interest, which demonstrated that the data were parametric. Little’s Missing Completely At Random (MCAR) test indicated that the data were missing at random (*p* > 0.05 [51]) and hence, missing data were removed from the following analyses. A series of repeated-measures ANOVA tests were conducted to investigate whether significant differences existed in the differences in scores of the variables of interest between conditions, to assess Hypothesis 1. For Hypothesis 2 and Hypothesis 3, a series of Simple Linear Regressions were conducted. The a priori sample size calculation in G*Power [52] assumed that the data collected will be non-normally distributed. However, the data collected were normally distributed. Therefore, post hoc analyses were conducted in G*Power [52] to determine the power of the statistical tests. The power for the repeated measures ANOVA tests was small (range: 0.05–0.28) and for the linear regressions was small to medium (range: 0.09–0.44) when the actual data outcomes were used.

## 3. Results

### 3.1. Participant Characteristics

The relevant emotional profile of the participants is presented in Table 1. In general, participants reported engagement in healthier emotion regulation strategies (i.e., cognitive reappraisal instead of expressive suppression), low levels of alexithymia, and high levels of self-efficacy. Additionally, participants scored higher on the conscientiousness scale than on openness, agreeableness, and extraversion.

### 3.2. Hypothesis 1—Differences between Thermal Conditions

**Figure 3:**
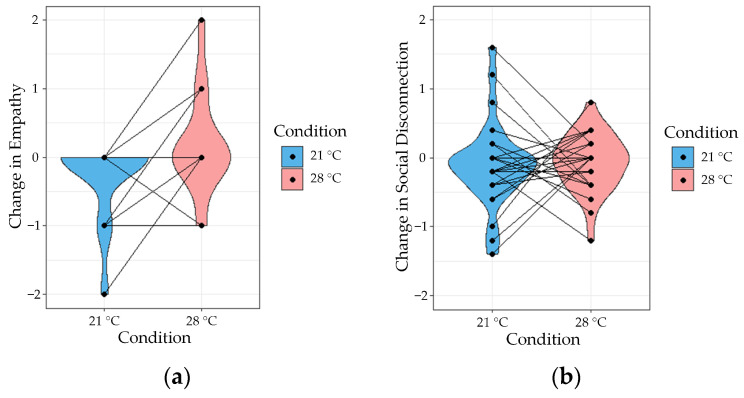

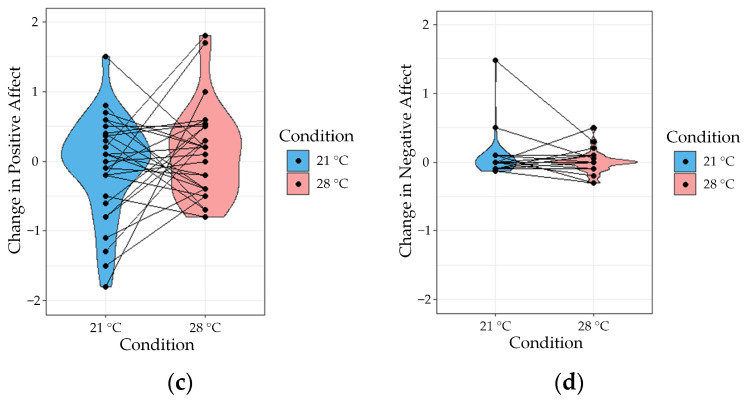
Changes reported in (**a**) empathy, (**b**) social disconnection, (**c**) positive, and (**d**) negative affect for the cool (21 °C) and warm (28 °C) condition.

The raw data for Hypothesis 1 are presented in Table 2 and Figure 3. Four one-way repeated measures ANOVA tests were conducted to identify differences between conditions on the reported changes in empathy, social disconnection, positive and negative affect levels after thermal exposure. The results indicated that there was a significant difference between conditions in the changes reported in empathy (*F*(1, 24) = 5.37, *p* = 0.03, with an *η*^2^ = 0.126), demonstrating that more empathy was reported after being exposed to the warm condition. However, no significant differences were reported for changes in social disconnection (*F*(1, 28) = 0.19, *p* = 0.67, with an *η*^2^ = 0.003), changes in positive affect (*F*(1, 29) = 1.86, *p* = 0.18, with an *η*^2^ = 0.029) and changes in negative affect (*F*(1, 29) = 1.39, *p* = 0.25, with an *η*^2^ = 0.017) between conditions.

### 3.3. Hypothesis 2—Effect of Thermal Sensation

**Figure 4:**
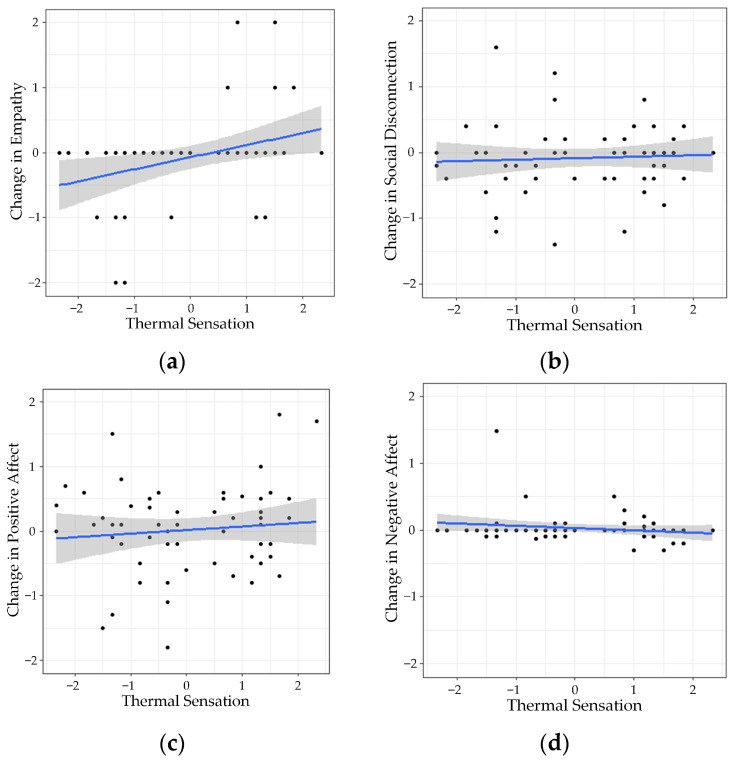
Changes reported in (**a**) empathy, (**b**) social disconnection, (**c**) positive, and (**d**) negative affect according to mean thermal sensation votes of the day. The blue lines represent the regression line of the data points and the grey area around it represents the confidence intervals.

The raw data for Hypothesis 2 are presented in Figure 4. Four linear regression analyses were conducted to identify the effect of thermal sensation on the changes in reported empathy, social disconnection, positive and negative affect levels after thermal exposure. The results indicated that the mean thermal sensation vote of the day had a significant positive effect on changes in reported empathy levels (*F*(1, 54) = 7.015, *p* = 0.01, with an *R*^2^ = 0.115), demonstrating increases in reported empathy when the environment was sensed as warmer rather than cooler. No significant effects of mean thermal sensation of the day were found on changes in reported social disconnection levels (*F*(1, 58) = 0.1762, *p* = 0.68, with an *R*^2^ = 0.003), positive affect (*F*(1, 59) = 1.169, *p* = 0.28, with an *R*^2^ = 0.019) and negative affect (*F*(1, 59) = 2.07, *p* = 0.16, with an *R*^2^ = 0.034).

### 3.4. Hypothesis 3—Effect of Mean Skin Temperature

**Figure 5:**
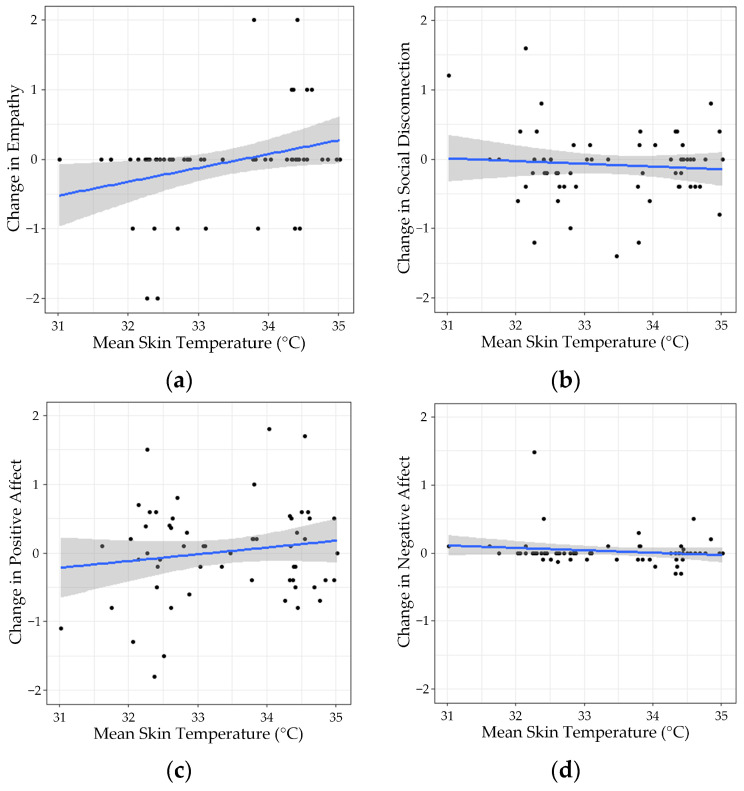
Changes reported in (**a**) empathy, (**b**) social disconnection, (**c**) positive, and (**d**) negative affect according to the mean skin temperature of the day. The blue lines represent the regression line of the data points and the grey area around it represents the confidence intervals.

The raw data for Hypothesis 3 are presented in Figure 5. The analyses demonstrated that the mean skin temperature had no significant effects on empathy (*F*(1, 6) = 0.53, *p* = 0.89, with an *R*^2^ = 0.81), social disconnection (*F*(1, 58) = 0.38, *p* = 0.54, with an *R*^2^ = 0.007), positive affect (*F*(1, 59) = 1.68, *p* = 0.20, with an *R*^2^ = 0.03) and negative affect (*F*(1, 59) = 1.57, *p* = 0.22, with an *R*^2^ = 0.03).

## 4. Discussion

The goal of this study was to investigate how empathy, social disconnection, and positive and negative affect are influenced by different thermal conditions at work. The results indicated a significant difference between the pre and post exposure change in empathy reported by participants in the cool vs. the warm indoor temperature environment in the simulated office space. Participants reported higher levels of empathy after being exposed to the warm condition (28 °C) than after being exposed to the cool (21 °C) simulated work environment. A similar pattern was observed for the positive affect, although the results did not reach significance. No significant differences were reported between the warm and cool condition in terms of negative psychosocial aspects, such as social disconnection and negative affect. Additionally, a positive effect of thermal sensation on empathy was found suggesting that perceiving the environment as warmer rather than cooler can lead to increases in empathy levels. Lastly, mean skin temperature showed no significant effect on the variables of interest. These results suggest that warmer thermal exposures during the day at work could influence how much empathy we are expressing towards other people at the end of the working day. This association could be mediated by perceived thermal sensation. However, further research with a larger sample is needed to investigate these mediational pathways.

These results support the positive effect of temperature on psychosocial aspects previously demonstrated by studies suggesting that there is a shared neural network between physical and social warmth [15,17,18,19]. However, effects were reported in empathy due to changes in subjective thermal sensation and not due to the changes reported in the objective skin temperature. Consequently, one could argue that our thermal perception of the environment has a more important role in regulating our psychosocial aspects than actual physical warmth during work, although these effects were very modest. This effect could be due to the fact that people associate higher temperature conditions with the opportunity to go out and be involved in more social activities [25,53]. However, other variables, for example the availability of other people to go out, can further influence the outcomes, suggesting the presence of combined effects, which supports the modest direct effect of the thermal perception on psychosocial variables. Another explanation for this observation could be that physical warmth could also be regulated by how socially connected we feel in general, as is supported by the Human Penguin project [54], which found associations between social warmth and core body temperature. However, it is important to consider that previous thermal exposure has been found to influence thermal perception [55]. Consequently, the effect of the specific temperature levels used in this study cannot be generalized to other climatic conditions. Future studies could replicate the current study to observe if these associations remain valid under different climates. Similarly, the short-term exposure to these increased temperature conditions might not reflect individuals’ reactions after long-term exposures to increased temperatures since short-term exposures do not reflect the ability of an individual to adapt to the situation. Future studies could assess empathy and other psychosocial variables through longitudinal studies using ecological momentary assessment in order to explore the long-term effects of cool and warm temperatures and potential adaptation processes to the thermal conditions.

In contrast to previous studies suggesting a shared neural network between physical and social warmth, no significant differences were reported in changes in social disconnection between the warm and cool condition. One explanation could be the lack of power in the analysis to demonstrate this effect. Another explanation could be that social connection is fostered since birth from the mother–child relationship and it is proven to last until adulthood [56]. Therefore, longer exposures to different temperatures might be needed to observe any changes in feelings of social disconnection. The emotional profile of the participants supports the latter hypothesis, since the ability to engage in adaptive coping strategies, as reflected in the emotional profile of the sample, could suggest that the participants had/have a secure attachment with their mothers [57]. Therefore, long-established feelings of social connection might be difficult to change by short-term temperature exposures. Future studies could investigate the influence of the thermal environment on psychosocial variables of interest in a sample with insecure attachment or in a longitudinal design with a securely attached sample.

Furthermore, no significant differences between conditions and no significant effects of thermal sensation and skin temperature were found on negative affect. These findings contradict previous evidence suggesting a correlation between thermal conditions and negative affect [58]. One possible explanation is the absence of probing variables such as pictures and cognitive tasks in this study. The probing variables used in previous studies could have triggered feelings of discomfort, as suggested by the authors, driving the observed change in negative affect. Another explanation could be that participants were more willing to report positive changes than negative changes [59], leading to a reported greater effect of the conditions on the positive psychosocial factors. Alternatively, the adaptive coping strategies generally employed by the participants could have helped in regulating any potential negative affect feelings arising from the thermal conditions.

The findings of this study suggest that increases in indoor temperature could lead to positive psychosocial changes. Therefore, one can argue that warmer indoor temperature conditions at work, as a consequence of climate change, would have positive effects on the empathic responses of the employees instead of being perceived as an environmental stressor for them. These outcomes provide a great basis to extend future research on potential resilience factors of climate change within a work environment setting. Increases in empathic responses might moderate some of the negative outcomes of climate change, maintaining a form of resilience within the employees caused by the increased temperature conditions. Future studies should: (i) replicate current findings using a larger and more divers sample, (ii) investigate how increases in empathic responses could help in reducing the effects of climate change on physical and mental health, and (iii) explore how changes in empathy could be considered in future prediction models and preventive strategies. Additionally, the observed lack of association between temperature and negative psychosocial aspects could be investigated longitudinally, to observe whether negative psychosocial aspects require longer exposures to show any changes, or if moderating effects of other variables are masking the effects of the thermal conditions on social disconnection and negative affect.

The findings of this study should be considered in light of its limitations. Although the study followed a repeated-measures study design, which requires smaller sample sizes to achieve significant power, the power of the analysis was still low to indicate any smaller effects of the conditions on the variables of interest. Additionally, the small sample size of the study limits the generalizability of the study outcomes only to individuals with similar characteristics. Due to the lack of previous evidence on the effects of indoor air temperature on empathy, the study followed an exploratory design. Therefore, causal effects cannot be inferred. The lack of momentary assessment in terms of empathic responses in previous studies led to the use of a non-validated measure to investigate changes in empathic responses, and therefore, future studies are needed to confirm the reliability of these findings. A replication of the study investigating changes in empathic responses using ecological momentary assessment can be enlightening. Furthermore, other moderating factors might have influenced the association between temperature conditions and levels of empathy, such as the relationships formed between the participants in the room and the content of the activities performed. Future studies could consider replicating this study on a larger sample, while controlling for the activities performed during the exposure and the level of social connectedness developed between the participants in the room. Nevertheless, despite the limitations, the study provided important preliminary evidence of the psychosocial impact of rising temperatures as an outcome of climate change, which was lacking within the literature.

## 5. Conclusions

In conclusion, the current study investigated the effect of a cool (21 °C) and a warm (28 °C) thermal environment in a simulated office laboratory on psychosocial aspects, such as empathy, social disconnection, and positive and negative affect. Our findings support there being differences between the two thermal conditions regarding positive psychosocial aspects (i.e., empathy and positive affect), but not regarding negative psychosocial aspects (i.e., social disconnection and negative affect). The changes observed were influenced by subjective thermal perception. These preliminary findings could potentially have important implications in enhancing our understanding on how future increases in temperatures could influence the psychosocial experiences of the population, which can help in the early establishment of relevant resilience strategies. Further research is needed to replicate these findings in a larger sample and in a more longitudinal study design using ecological momentary assessment.

## Figures and Tables

**Table 1:** Participants’ personality, emotion regulation, alexithymia, and self-efficacy characteristics.

	*N*	Mean	SD	Interpretation
BFI-10: Extraversion	30	6.97	1.79	Moderate levels of extraversion
BFI-10: Agreeableness	30	7.00	1.46	Moderate levels of agreeableness
BFI-10: Conscientiousness	30	7.80	1.13	High levels of conscientiousness
BFI-10: Openness	30	7.53	1.76	Moderate levels of openness
ERQ: Cognitive Reappraisal	30	29.97	5.42	Moderate use of cognitive reappraisal
ERQ: Expressive Suppression	30	13.63	5.47	Low use of expressive suppression
ERQ: Total	30	48.33	8.71	Moderate levels of emotion regulation
TAS: Total	29	42.79	8.09	Low levels of alexithymia
SWE: Total	30	30.93	3.06	High levels of self-efficacy

**Table 2:** Average pre/post exposure changes in the variables of interest under the two temperature conditions (21 °C and 28 °C).

	*N*	Mean at 21 °C	Mean at 28 °C
Pre/post exposure change in empathy	30	−0.28	0.19
Pre/post exposure change in social disconnection	30	−0.09	−0.07
Pre/post exposure change in positive affect	30	−0.08	0.21
Pre/post exposure change in negative affect	30	0.06	−0.01

## Data Availability

The dataset will be provided by the authors upon reasonable request.
